# Toward High-Performance Coatings for Biomedical Devices: Study on Plasma-Deposited Fluorocarbon Films and Ageing in PBS

**DOI:** 10.3390/ma3031515

**Published:** 2010-03-02

**Authors:** Servaas Holvoet, Pascale Chevallier, Stéphane Turgeon, Diego Mantovani

**Affiliations:** Laboratory for Biomaterials and Bioengineering, Department of Materials Engineering, University Hospital Research Center, Laval University, Quebec City, QC, G1K 7P4, Canada; E-Mails: servaas.holvoet@crsfa.ulaval.ca (S.H.); pascale.chevallier@crsfa.ulaval.ca (P.C.); stephane.turgeon@crsfa.ulaval.ca (S.T.)

**Keywords:** ageing, oxidation, plasma polymers, fluorocarbon films, XPS, FTIR, AFM

## Abstract

High performance coatings tailored to medical devices represent a recognised approach to modulate surface properties. Plasma-deposited fluorocarbon films have been proposed as a potential stent coating. Previous studies have shown promising adhesion properties: the 35 nm-thick film sustained plastic deformation up to 25% such as induced during the clinical implantation. In this study, the compositional and morphological changes of plasma-deposited fluorocarbon films were examined during ageing in a pseudo-physiological medium, a phosphate buffer solution (PBS), by angle-resolved XPS, FT-IR data and AFM images. The evolution of the ageing process is discussed: defluorination and crosslinking yielded an oxidized protective top layer onto the films, which showed further degradation.

## 1. Introduction

Thin films have been developed over the last five decades in order to improve surface properties of biomaterials for biomedical use. A major problem in biomaterial research is the interfacial biocompatibility between synthetic prosthesis and living tissue, and the best approach seems to be the modulation of tissue/biomaterial interface by modifying the synthetic graft surface properties in order to achieve significant improvement of its interaction with blood and living tissues [1]. One type of such modifications is the deposition of a thin interfacial biocompatible film. Deposited thin films should have inert properties such as biocompatibility, stability in biological environment and specific properties depending on their function. For example, several coatings have been investigated for knee and hip prostheses in order to increase their resistance to abrasion and improve their lubrication [2]. For cardiovascular biomaterials, efforts have focused on developing surfaces with improved human blood compatibility: for instance phosphorylcholine [3,4,5,6] and heparin [7,8,9,10,11,12,13] coatings for improved hemocompatibility, polyethylene glycol (PEG) acting as non-fouling surfaces [12,14,15,16,17,18] and bioactive coatings such as drug delivery systems [12,13,19,20,21,22]. Moreover in the field of stents, which are thin metal wire mesh tubes placed inside partially blocked arteries to prevent the obstruction of blood flow and to act as internal scaffolding [23], mainly made of 316L stainless steel, it has previously been shown that metals can be effectively protected against corrosion by the deposition of thin polymer coatings [24,25,26,27]. Stent corrosion might lead to ion release in the physiological environment, and induces further complications at clinical levels [28]. Possible corrosion products include chromium and nickel, two elements classified as carcinogenic by the International Agency for Research on Cancer (IARC) [29]. The Food and Drug Administration (FDA) has recently provided guidance for the stent industry to evaluate the safeness and effectiveness of coated and drug-eluting stents [30]. Adhesion, barrier effectiveness, durability and stability of the coating are only a few requirements that need to be assessed to demonstrate the efficiency of the coating [31,32].

In order to deposit thin films, several approaches can be used, such as: dip, spin or spray coating, chemical vapour deposition and plasma polymerization. Regardless of the application, the coating should have adequate properties such as good adhesion onto the material and stability in the biological environment. Films deposited by dip, spin, and spray, as well as chemical vapour depositions have exhibited lack of adhesion when submitted to deformation, friction or shear stress such as induced by the blood flow. Indeed, delamination or cracks in these films were observed [33]. However, the plasma polymerization process should induce better adhesion between the polymeric layer and the substrate due to the initial activation of the surface.

In this context, to improve the long-term performance and safeness of stainless steel stents, a multi-step process was previously developed in our laboratory in order to isolate the stainless steel surface from the body fluid by depositing an ultra-thin uniform and cohesive (~35 nm) plasma polymerized fluorocarbon (Teflon-like) coating [34]. Results showed that the coatings resisted to plastic deformations up to 25%. Moreover, in order to abide to strict FDA regulations, the coating must also be stable after implantation. In an attempt to assess the stability after implantation, the aim of this work was to investigate the stability of the plasma polymerized thin coating deposited on 316L stainless steel substrate using a pseudo-physiological ageing medium. The effect of 25% plastic deformation on stability and ageing characteristics of the fluorocarbon coating were also verified.

The pseudo-physiological ageing medium used in this work was a phosphate buffered solution (PBS) chosen for its degree of acidity similar to physiological pH (~pH 7.4) and for its ionic strength and composition similar to that of blood [35]. Finally, the absence of non-electrolytes such as sugars and/or proteins (in contrast with e.g. Hanks medium) prevents the PBS medium from masking the chemical composition of the surface due to deposition of the latter species.

After different periods of immersion in PBS buffer, characterization of morphology and chemical composition of the coating was performed by Atomic Force Microscopy (AFM), Fourier-Transform Infra-Red spectroscopy (FT-IR) and X-ray Photoelectron Spectroscopy (XPS).

## 2. Results and Discussion

### 2.1. Ageing of coated samples

**Figure 1:**
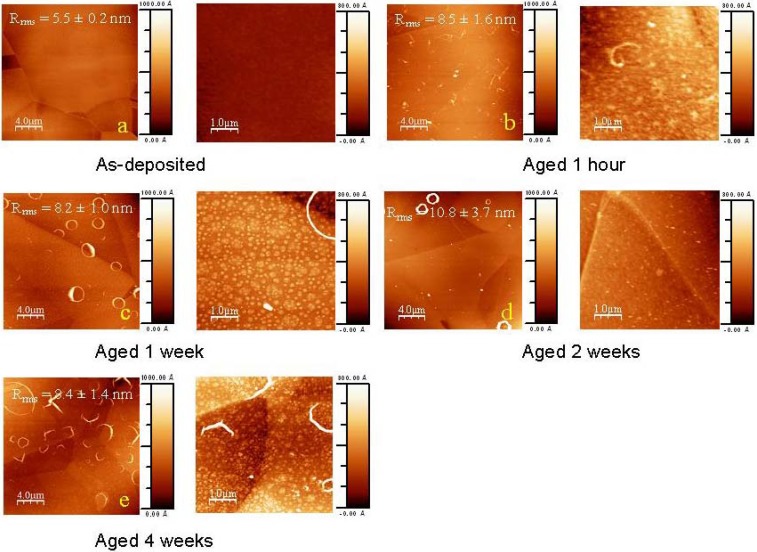
Tapping mode AFM micrographs 20 × 20μm^2^ for flat samples at different time points of ageing: (a) as-deposited, (b) aged 1 hour, (c) aged 1 week, (d) aged 2 weeks, (e) aged 4 weeks and zoom 5 × 5 μm^2^ of each.

The ageing effect on the flat coated sample morphology was observed by AFM (Figure 1). Initially, the as-deposited coating was fairly smooth (R_rms_ = 5.5 ± 0.2 nm, Figure 1a), with the stainless steel grain boundaries clearly visible. In general, no deposits of salts from the pseudo-physiological medium were observed. Two different surface phenomena have occurred during the first week of ageing. Immediately after incubation into the liquid (after 1 hour), linear, curly structures of a few micron in length could be observed onto the surface, as presented in Figure 1b. Their presence has a limited impact on the surface roughness, which increased from 5.5 ± 0.2 nm to 8.5 ± 1.6 nm. These polymeric strands have probably detached from the outermost surface, but due to their non-solubility in the ageing medium, they remained adhered to the fluorocarbon film. Moreover, as illustrated in Figure 1c, many of these disordered short polymeric chains coalesced during the first week into a pattern of organised, almost perfectly circular structures by self-assembly, in this way minimizing the surface energy. These circular features possessed diameters of approximately 1–2 micron and a height of 30 nm. Surface roughness had stayed rather constant (8.2 ± 1.0 nm). According to the corresponding phase image (not shown), the phase shift of the probe tip was higher on the circles than on the other areas, indicating that those structures could be softer or more hydrophilic than the average surface. At that time, namely week one, the 5 × 5 µm^2^ image revealed that, besides the micron-sized circles, the entire surface was covered with round nano-scaled protrusions (Figure 1d). The latter appeared to be more or less circularly shaped and hill-like, varying between 200 and 500 nm in diameter and around 5 to 10 nm in height. Moreover, small peaks (intense spots) could be observed close to the centre of the round features. These round features are believed to be the sites of nano-pinhole defects, present on the coating after plasma deposition process [36], which enabled the buffer to seep into the coating and initiate the degradation process.

Therefore, water progressively infiltrated the polymeric coating, creating the protrusions which then grew. For specimens aged for 2 weeks, the amount of micron-sized structures decreased significantly (Figure 1d), and moreover, the small nano-scale protrusions had smeared by joining. Thus, the coating was swelled irregularly and the surface roughness has slightly increased, but the difference was not statistically significant (due to the high standard deviation of 3.7 nm). After 4 weeks of ageing, the surface appeared to be covered with remaining degradation products (Figure 1e). Moreover, at higher magnification, new nano-scaled protrusions appeared possibly due to corrosion of the metallic components at the interface below the nano-pinholes [37].

**Figure 2:**
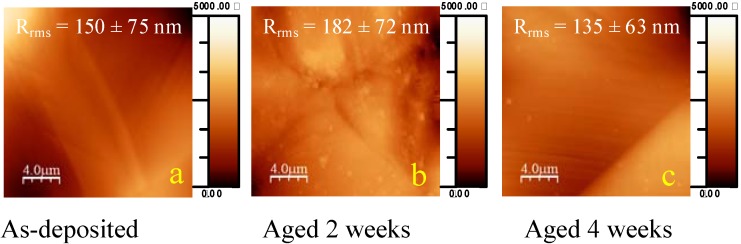
Tapping mode AFM micrographs for deformed samples at different time points of ageing: (a) as-deposited, (b) aged 2 weeks, (c) aged 4 weeks.

After deforming an as-deposited coating, the surface roughness had increased drastically from ~10 nm to ~150 nm, due to the presence of slip bands induced by plastic deformation [34] (Figure 2a). In contrast to flat specimens, deformed films incubated for periods varying from a few minutes up to one week presented no differences in surface roughness. Some remains of curly structures could be observed on the one week aged sample, similar to what was observed on the 4 weeks aged flat sample. After 2 weeks, all that remained on the deformed specimens were nano-sized protrusions (globular degradation clots) which became very scattered after 4 weeks.

XPS analysis of both non-aged and aged samples yielded C, F and O signals, without the presence of the underlying metallic components Fe, Cr and/or Ni. In addition, no deposition of components from the ageing medium such as Na, K, chlorides or phosphates was observed. XPS analyses were performed at two different angles in order to have a better idea of the ageing process induced on the fluorocarbon film at different depths (Figure 2). Indeed, due to the electron attenuation length λ of photoelectrons for C (1s), O (1s) and F (1s), the depth from which 95% of the detected XPS signals originated were estimated to range between ~4.5–6.0 nm for normal emission (at 90°) and ~1.0–1.5 nm for grazing emission (at 15°) [38,39].

**Figure 3:**
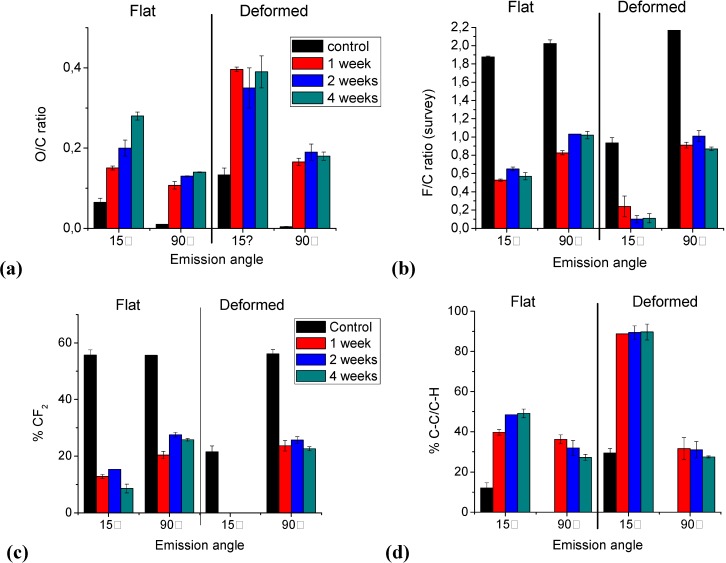
Ratios of (a) O/C and (b) F/C deduced from XPS survey data and XPS C(1s) high resolution data for (c) % CF_2_ and (d) % C-C/C-H as a function of ageing time, measured at two emission angles (15° and 90°) for flat and deformed samples.

The O/C survey data, presented in Figure 3a, indicated that the composition of the fluorocarbon coatings varied both with depth and ageing time. XPS detected some oxygen in the as-deposited plasma polymers, although at a low concentration (O/C = 0.065), probably due to reactions with atmospheric oxygen and water vapour. Different authors have shown that for plasma-deposited films, oxygen is incorporated in the XPS probe depth within very short times upon contact with air [38,39,40,41]. In this work, the detected oxygen may analogously be assigned to reaction between long-lived radicals trapped in the fluorocarbon coating and in-diffusing atmospheric O_2_. This initial reaction was diffusion-limited and concentrated in the surface outermost layers. An identical XPS experiment at normal emission pointed out the absence of oxygen deeper into the material. Thus by the time that the initial analyses were performed, some oxidation at the surface had already taken place.

Thereafter, two different trends could be observed for the O/C ratio upon ageing in PBS buffer. At the surface (θ = 15°), the oxygen content doubled after one week, and continued to increase with ageing time. Deeper down (θ = 90°) an analogous increase in O/C ratio was monitored after one week, however no significant variation in this value was monitored for higher ageing times, indicating selective oxidation reactions in the top layers of the coating.

Simultaneously, when interpreting F/C survey data (Figure 3b), a high degree of defluorination was observed. The F/C value for an as-deposited film (F/C ~2), which was rather constant for the entire film thickness, dropped to ~0.5 in the top layers and ~1.0 for deeper regions after 1 week. Upon further ageing, however, these values remained rather constant. Note that the magnitude of the decrease in the F/C ratio was higher than the increase of the O/C ratio. Thus some of the observed defluorination with ageing may be attributed to replacement by oxygen, but a large portion of this decrease could only be explained by other degradation mechanisms.

The percentages of the various structures present in an as-deposited film, measured at normal emission, were determined by curve fitting of the C (1s) signal to be 17.5% (CF_3_), 55.6% (CF_2_), 13.1% (CF), 13.9% (C-CF_x_) and 0% (C-C/C-H). The same information obtained at grazing emission revealed the presence of ~12% C-C/C-H components, which could indicate surface crosslinking in some extent. When focusing on the fluorinated carbon species, CF_3_ and CF_2_ specifically, a drastic drop was observed after one week of ageing (Figure 3c; CF_3_ is not shown). Thereafter, the composition of the coating did not vary much anymore, in analogy with the observed F/C survey data. A decrease of ~10% CF_3_ and ~45% CF_2_ components was observed at the top layers, but these values increased slightly when the measurements were performed at deeper regions. In addition, a large amount of non-fluorinated C-C (and C-H) species had formed into the coating, rising up to ~50% (Figure 3d). CF_3_ and CF_2_ components are known to contribute to a coating consisting of relatively high molecular weight, linear polymer chains, without high degrees of crosslinking and/or unsaturated structures. The important loss in the latter, combined with C-C as new and major component, indicated a change in the chemical structure of the coating, which upon ageing had degraded into a fluorine-poor material, probably with a highly crosslinked character.

After sample deformation, XPS analyses exhibited an uptake in oxygen (Figure 3a). Indeed deformed samples displayed an O/C ratio of around 0.13 in the top layers compared to a flat as-deposited specimen (O/C ratio ~0.065). This result indicated that surface oxidation probably occurred during the deformation, which can be explained by the fact that the deformation process was carried out in open atmosphere. Thereafter, oxidation occurred after incubation, as observed by a large increase of O/C after one week (Figure 3a) as well at 15° and 90°. After 1 week onto deformed samples, the O/C ratio remained constant upon further ageing for the top and deeper layers (Figure 3a). Note that this increase in O/C for deformed samples is higher compared to the flat samples discussed earlier. Whereas the F/C ratio measured at grazing angle for flat specimen decreased from ~1.9 to ~0.5, the initial value before ageing amounted to ~0.9 for a deformed specimen, dropped to ~0.2 after 1 week and to ~0.1 after a month (Figure 3b). Deeper however, an identical F/C value was measured for both flat and deformed coatings before and after ageing. It was evident that the plastic deformation caused a top layer oxidation and defluorination, which further influenced the characteristics of the ageing process. Corresponding to the drop in fluorine in this region was again the drastic loss in CF_3_ and CF_2_ moieties. At 15° detection, these fluorinated carbons could not be detected any more after ageing (Figure 3c). The only carbon functionalities left in the top layers were C-CF (~10%) and C-C/C-H (~90%) (Figure 3d), approaching those of an amorphous carbonaceous film on top. Deeper (90°), CF_2_ and C-C/C-H remained rather constant after 1 week of ageing: from 56% after deformation to 25% (Figure 3c) and from 0% to 30% respectively (Figure 3d). Again, no significant variation in both surface and deep composition was observed when the samples were aged longer than a week, which was consistent with the ageing data obtained for flat samples.

**Figure 4:**
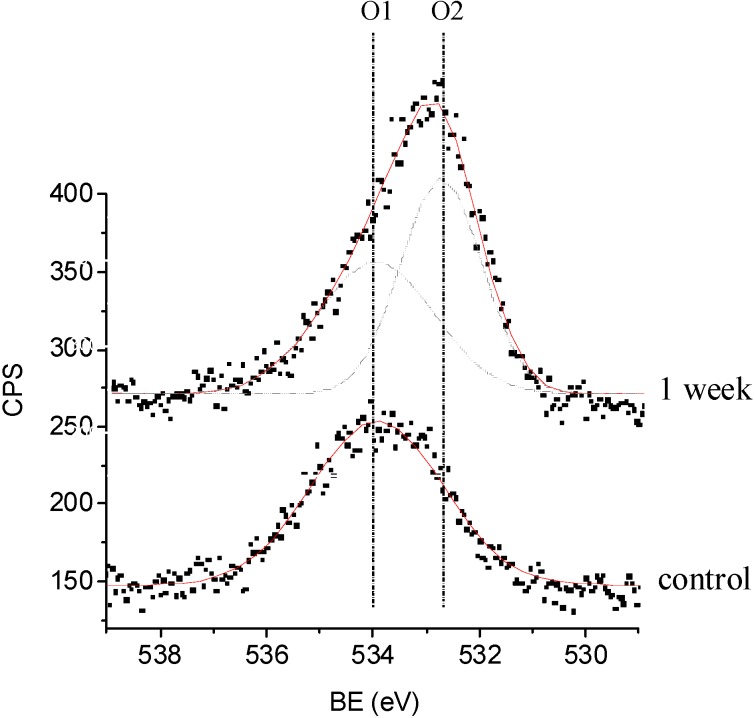
Representative XPS O (1s) spectra obtained from flat as-deposited and flat aged (1 week) samples, including curve fits.

To have a better understanding of the ageing process, high resolution XPS of O (1s) has also been acquired on an as-deposited sample and on a one-week-aged coating. The range of binding energy (BE) values determined for the two O (1s) components was 532.8–530.2 eV for component O1 and 534.1–534.5 eV for O2 (Figure 4). The former value is typical for O (1s) signals measured on oxidized hydrocarbon-based plasma polymers where a range of different carbon oxygen functionalities (e.g. alcohols, ethers, carbonyls) contribute to the signal. The binding energy of the second O (1s) component, which was unusually high, must have arised from shifts induced by the presence of fluorine. In literature, analysis of a perfluoropolyether reference material yielded a BE of the ether oxygen (CF_2_-O-CF_2_) of 535.8 eV [39]. Because the O (1s) BE of an aliphatic ether is known to be 532.6 eV [42] and assuming that each neighbouring CF unit shifts the O (1s) BE with an equal amount, the BE of a CF_2_-O structure adjacent to a non-fluorinated carbon could thus be estimated to be 534.2 eV [43]. So probably, the high-energy O (1s) component originated mainly from oxygen which had two to three fluorine atoms on adjacent carbons.

For a non-aged coating, all incorporated oxygen had bound between fluorinated carbons, as no shoulder towards the lower binding energy region could be observed. After 1 week of ageing, around 65% of the oxygen originated from hydrocarbon moieties. No significant variation in this latter value was monitored for longer ageing times (not shown). This is in agreement with the F/C trends discussed earlier, where the major degradation took place within the first week.

**Figure 5:**
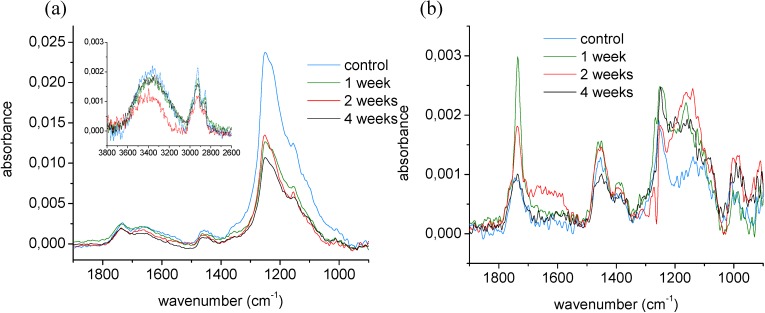
FT-IR spectra (ATR mode) of (a) flat samples and (b) deformed samples at different periods of ageing.

The structure of the entire thickness of plasma-deposited coatings was also investigated by Infrared spectroscopy (maximum analysis depth of 1 μm *versus* the coating of ~35 nm). Figure 5 presents the FT-IR spectra of non-deformed fluorocarbon-like thin coatings before and during their immersion in PBS buffer. The molecular vibration assignment was performed according to the literature [43,44]. The FT-IR spectra, which were not normalized in order to observe variations in the surface density of the coating, could be divided in four important features. First, the most prominent feature is the broad absorbance band in the 950–1390 cm^-1^ region which could be assigned to CF_x_ (x = 1–3) or to C-O stretching modes. The presence of the CF_2_ functionality could be seen in all spectra upon ageing indicating that CF_2_ groups in the solid films were organized in an ordered manner through linear attachment. Second, there was a broad absorbance band from 1500 to 1800 cm^-1^ associated with C=C and/or C=O vibrations and O-H bending from absorbed water. Third, a feature located between 2790–3050 cm^-1^ could be attributed to CH_x_ (x = 1–3) vibration modes. Finally, there was a broad band between 3050 and 3600 cm^-1^ which is characteristic for O-H stretching from alcohol functionalities and/or absorbed water.

In Figure 5a, it could be noticed that the broad band in the 1000–1400 cm^-1^ region became less intense after the first week of ageing for the flat samples. However, from the second to the fourth week of ageing, the intensity of the spectra in this area did not show any significant variation. The initial decrease of intensity might be related to a loss of fluorine components from the coating, as demonstrated by XPS earlier. After integration of the FT-IR 1000–1400 cm^-1^ region, the ratio between the peak areas after and before one week of ageing amounted to ~0.56. Analogously, the ratio of the F/C values obtained by XPS survey analysis earlier (at 90° emission angle), was ~0.41. In addition, the constancy of the degree of fluorination for higher ageing times measured with XPS was in this way confirmed by the FT-IR data. This band did not become broader upon ageing, which could be an indication that the film matrix kept its well-ordered character and did not become more amorphous with time. In our case, it was difficult to differentiate the oxy bands (1272–1103 cm^-1^) from those due to CF_x_ (1400–1000 cm^-1^). However, as XPS data demonstrated earlier that oxygen content in the bulk was very small compared to fluorine content, this band decrease was fully ascribed to defluorination and not to oxidation.

FT-IR trends differed slightly for plastically deformed coatings. First of all, the absorbance intensity was much lower compared to the analyses on flat samples, indicating a thinning of the coating due to plastic deformation. Secondly, where similar spectral features could be assigned in the FT-IR spectra of deformed samples, the intensity of some specific bands was different. The CF_x_ band intensity was very low compared to other bands and moreover, displayed little variation upon ageing. However, an increase in absorbance for the oxy bands (1272–1103 cm^-1^) could be observed. The loss in fluorine, as detected by XPS (at 90°) earlier, was probably compensated with an increase in oxygen functionalities. When focusing onto the C=O stretch in the 1750 cm^-1^ region, its intensity increased greatly during the first week of ageing, but dropped again thereafter, reaching the initial intensity level after one month, as presented in Figure 5b. So during the first week of ageing a significant amount of oxidized functionalities (containing carbonyl moieties) was incorporated into the film. After the first week, this fraction rich in polar components was apparently dissolved out of the coating.

### 2.2. Mechanisms of the ageing process of fluorocarbon films

Experimental results exhibited that the ageing processes were not the same for flat and deformed samples, indicating different mechanisms.

#### 2.2.1. Ageing of non-deformed samples

During the **first week** of incubation, buffer had penetrated the top layers and had diffused into the bulk of the coating, as visualised on AFM images by the round protrusions around the nano-pinhole defects. The phenomenon of the micron-sized circular features, observed before the protrusions were present, was slightly more complex to elucidate. In the AFM micrograph obtained after one hour of incubation, assembled polymeric chains were stuck to the outermost surface. Earlier, XPS analysis of these top layers (at 15°) before ageing had demonstrated the outermost film to differ slightly in chemical composition from the layers underneath. As these top layers were slightly more oxidized (and more hydrophilic) than the underlying bulk film, buffer penetrated from the surface through this top nano layer. This top layer probably detached from the bulk and due to the non-polar character of the material (and as a consequence, its non-solubility into the ageing medium), the fluorocarbon polymeric material tried to minimize its contact surface with the liquid by curling up into circles. They would stay adhered onto the film surface until oxidation has proceeded in that extent, enabling dissolution from the surface thereafter.

In the bulk, the interaction of buffer with the fluorocarbon polymers resulted into the loss of CF_3_ and CF_2_ moieties, demonstrated by high resolution XPS analysis at 90°. In addition, FT-IR analysis over the entire thickness confirmed this phenomenon of defluorination.

The main difference in composition between the bulk and the outermost surface is that these latter trends were observed to be more extreme at the top layers. The high amount of C-C moieties indicated the formation of a thin, highly crosslinked, oxidized film onto the surface, mainly consisting of C-CO_x_ components, which was confirmed by curve fitting the O (1s) signal. Also in the work of Horie *et al.*, the presence of such C_x_F_y_O_z_ species was observed in aged plasma-deposited CF_x_ films and was proved by secondary ion mass spectrometry experiments [45].

**Figure 6:**
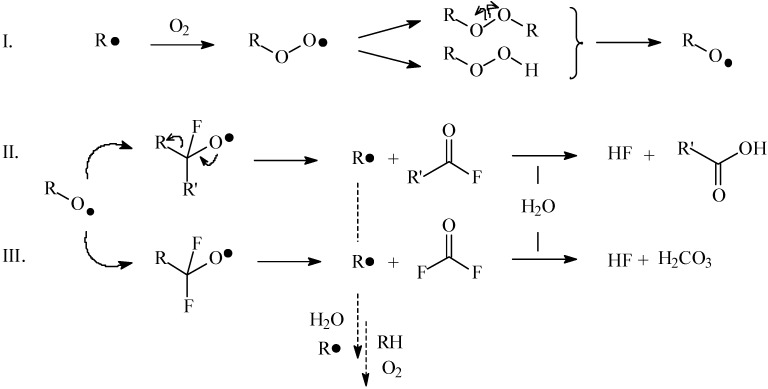
Schematic presentation of the defluorination pathway.

From a mechanistic point of view, the initially generated carbon-centered radicals were believed to react very fast with in-diffusing oxygen to form peroxy radicals (Figure 6, pathway I). These peroxy radicals, which are less reactive than carbon-centered radicals but nevertheless unstable, are known to combine with carbon-centered radicals to form peroxides, which can subsequently dissociate to form perfluoro-alkoxy radicals [39,46]. Consequently, depending on the nature of the neighbouring carbon, *i.e.,* a secondary or primary carbon, two different degradation pathways could be assumed. In a first case, CF-based perfluoro-alkoxy radicals could convert into acid fluorides and a new carbon radical (pathway II). Acid fluorides however tend to hydrolyse, yielding carboxylic acid groups accompanied with the release of hydrogen fluoride. The postulated release of HF is consistent with the reduction of the fluorine content observed by XPS and the reduced intensity of the FT-IR C-F bands. Furthermore, CF_2_-based perfluoro-alkoxy radicals (-CF_2_-O**·**) could decay by releasing carbonyl fluoride (CF_2_O), in the process again generating a new carbon-centered radical (pathway III). This reaction also leads to a loss of fluorine, in particular CF_2_ groups, as observed by XPS. The simplified mechanistic pathway of defluorination is schematically recapitulated in Figure 6. The newly generated carbon-centered radicals can subsequently recombine (R**·**), abstract a proton (RH), or react with water/oxygen with the formation of oxygen-centered radicals.

Thus the first pathway (by secondary carbons, pathway II) yields a variety of oxidized carbon functionalities with a low molecular weight, due to chain scission. A similar oxidation and low-molecular-weight oxidized material (LMWOM) formation process was reported in moisture uptake studies on plasma-deposited fluorocarbon films [47]. In this work however, the FT-IR signal did not show much variation in intensity, indicating that this mechanism was probably not the major reason of degradation. As as-deposited films have shown to contain high fractions of linearly polymerized CF_2_ chains, the second degradation reaction (on primary carbons, pathway III) was more likely to occur. This mechanism, corresponding with consecutive CF_2_ losses, resulted in primary carbon-centered radicals which could recombine and crosslink, in this way responsible for the highly-crosslinked and fluorine-poor nature of both the bulk and the uppermost layers.

The **second week** of ageing in PBS did not bring huge variations in both surface and bulk composition. Evidentially, during the first week, the coating had lost a significant amount of fluorine and, as a consequence, had become more hydrophilic. Because the film did not repel the buffer as much, a more uniform absorption of the buffer into the film was assumed, which made the protrusions smear out and disappear as observed in AFM. In addition, most micron-sized circular structures had dissolved.

XPS and FT-IR analyses exhibited that the bulk composition did not vary with increasing ageing time. A possible explanation for the constancy in bulk composition could be the following. Due to the high crosslink density at the surface, the permeability of the surface film for both oxygen and buffer had been substantially declined and as a consequence, the move of the oxidation front inwards the bulk had been slowed down and quasi stopped. This highly crosslinked top layer was thus assumed to act as a protective barrier against further bulk oxidation. This latter hypothesis was confirmed by a study of samples aged for **four weeks**. Both surface and bulk composition had stayed rather constant, as demonstrated by XPS and FT-IR, in favour of the idea of a protective top layer.

However on the AFM images, this highly crosslinked and polar top coating seemed to partially dissolve into the PBS ageing medium. This is in agreement with earlier work by Horie *et al.* who described the formation of RO_2_H groups (R= -CF_2_, -CF and -C) leading to the release of LWMOM from the plasma modified surfaces [45]. They demonstrated that non-water-soluble but ethanol-soluble LMWOM started to dissolve when the coatings were incubated into ethanol. The polarity of their films was not high enough to be soluble into aqueous media, as suggested by the difference in ethanol/water-rinsed and water-rinsed surfaces. Their films possessed O/C ratios around 0.1, whereas for our films these ratios have shown to mount up to ~0.3. This higher polarity, and the correlated water solubility, were only obtained after one month, and could explain why the protective layer started to dissolve into the PBS medium. Hence a direct correlation between the surface hydrophilicity (related to the F/C and O/C content) and the dissolution rate of the film was assumed. The protective top layer would appear only metastable.

#### 2.2.2. Ageing of plastically deformed samples

Similar trends were observed during an ageing study on deformed coatings, namely a fast defluorination in the surface. The mechanism of ageing however tended to differ from the one described for non-deformed samples. In general, the rate of degradation for deformed specimens appeared to be much higher.

Again, from one week on, no significant compositional variation could be distinguished upon further aging. Whereas on flat samples protrusions were visualized around nano-pinhole defects after one week, which were disappearing after two weeks, the absence of the latter onto the surface of deformed samples could be an indication that the rate of buffer absorption was now much higher and more uniform. From the very beginning of the incubation experiments, no variation in surface topography was observed. Coating degradation products in the shape of circular structures were demonstrated to be present already after one hour of aging on deformed samples. These fluorocarbon film fragments again minimized their contact with the buffer by fouling into globular adherent clots.

The key to a possible explanation for this phenomenon was assumed to be found in the initial composition of the control samples, namely the non-aged specimens before and after plastic deformation. Where an initial F/C around 2.0 was measured on the outermost layers of a flat control sample (with XPS at 15°), this ratio dropped to ~0.93 after the deformation. The plastic deformation, carried out in atmospheric conditions, had proved earlier to have no significant influence onto the coating bulk composition [34], however changed the chemical nature of the top nanometer layer on the film. Chain scission, accompanied with radical-based crosslinking reactions, caused a fluorine drop in the outermost layers. Indeed, ToF-SIMS experiments on these coatings after deformation proved earlier to be accompanied with polymeric chain scission [36]. In addition, atmospheric oxygen probably diffused in high quantities through the surface and oxidized the top film, in this way explaining the high O/C ratio for an as-deposited deformed coating (0.13 *versus* 0.065 for a flat film respectively). The deformation procedure thus created samples with an identical bulk composition, but with a thin layer of a highly polar, oxidized and fluorine-poor material on top. These changes in the surface chemical composition were believed to affect the ageing phenomena which were observed on deformed coatings. Moreover, due to the plastic deformation of the steel substrate, the thickness of the fluorocarbon film was decreased on high deformation areas (cf. FT-IR), making it easier for the ageing medium to reach and affect the entire bulk.

Also, the top layers of deformed coatings seemed to have a higher polarity, as observed by XPS surveys. The O/C ratio for a deformed sample reached a value of 0.4 already after one week of ageing, whereas an equivalent of one month of ageing was needed for flat samples to obtain similar oxygen content of 0.3. This was also confirmed in the FT-IR data by the presence of the carboxylic bands, in relatively high concentrations for deformed samples after one week. As before incubation, the outermost surface contained high amounts of crosslinked material (with secondary fluorinated carbons), the degradation pathway (Figure 6, pathway II) by CF-based perfluoro-alkoxy radicals was favoured, yielding a variety of oxidized carbon functionalities (especially acid groups, as confirmed by FT-IR). This could make the dissolution rate of a deformed coating in the PBS buffer increase, compared to a lower polarity protective barrier on flat samples. The carboxylic bands around 1750 cm^-1^ decreased in intensity with further ageing, indicating the continued dissolution of the top layers.

So, on deformed samples, a highly polar top coating was produced during the deformation procedure, which possessed a solubility in water high enough to start the dissolution process immediately from the beginning of the ageing experiments. For flat samples, this coating composition was obtained a few weeks later, in this way inhibiting the removal of the uppermost layer during the first month of ageing. Important insight was yielded into the behaviour of plasma-deposited fluorocarbon films in contact with a buffered medium, in both morphological as chemical point of view. The observation of a metastable protective barrier upon ageing is an interesting phenomenon, which could be useful in further optimisation of the anti-corrosive properties of the films.

## 3. Experimental Section

### 3.1. Materials

Disks with 12.7 mm diameter and 0.5 mm thickness were punched from 316L stainless steel plates (Goodfellow, Devon, PA, USA) with the following composition (wt %): Cr (16.00–18.00), Ni (10.00–14.00), Mo (2.00–3.00), Mn (≤2.00), Si (≤1.00), C (≤0.08), P (≤0.045), S (≤0.03) and Fe (balance). Before the plasma polymerization, disks were subjected to a pre-treatment described in detail elsewhere [48] consisting essentially of four steps: 1. ultrasonic cleaning; 2. electropolishing; 3. acidic dipping; and 4. pulsed H_2_ (Bocgaz, 99.999% purity) plasma etching. The plasma deposition was performed at 11 cm below the glow region in the same reactor as the H_2_ plasma etching in order to prevent any contact with air and reoxidation of the substrate between the plasma-etching step and the plasma polymer deposition [34]. The feed gases used during plasma polymerization were a mixture of 94% of C_2_F_6_ (Sigma-Aldrich, 98+% purity) and 6% of H_2_. The pulsed plasma deposition was performed with the following parameters: RF peak power input = 150 W (13.56 MHz); t_on_ = 5 ms; t_off_ = 90 ms; gas pressure = 0.93 mbar; total gas flow rate = 20 sccm; duration = 5 minutes.

### 3.2. Deformation method

The coated samples were plastically deformed up to 25% using a custom-made small punch test device mounted on a SATEC T20000 testing machine (Instrod, Norwood, MA, USA), as previously described [34]. All deformations were performed at room temperature at a displacement rate of 0.05 mm/s and a load of 2200 N. All subsequent analyses were performed on the topmost part of the deformed samples, where the 25% deformation had occurred.

### 3.3. Surface ageing

Ageing tests were carried out in PBS buffer (Dulbecco´s PBS, Sigma-Aldrich) in an incubator at 37 ± 2 °C for periods ranging from one hour till one month (1, 2 till 4 weeks). Each sample was placed in a high density polyethylene sample holder in order to expose only its coated surface to the buffer (0.636 cm^2^ and 0.319 cm^2^ for flat and deformed samples respectively). Before the ageing tests, sample holders and ageing medium were sterilized by autoclave in order to prevent bacterial propagation during ageing. Samples were manipulated with sterilized (70% ethanol) tweezers under a laminar flow hood. After ageing, samples were removed from their holders and were thoroughly rinsed with deionised water and finally wiped with dry particle-free compressed air. Two samples per ageing time were prepared; one for AFM, FT-IR and CA analyses and the other one for XPS analyses. Chemical structure and morphology of the coatings were analyzed less than 24 hours after the immersion period. Before analyses, samples were carefully stored under vacuum.

### 3.4. Characterization of the ultra-thin coating

Atomic Force Microscopy investigations were performed using the tapping mode of a Dimension^TM^ 3100 Atomic Microscope (Veeco, Woodbury, NY, USA) with an etched silicon tip (OTESPA^TM^, tip radius < 10 nm). One sample per period of ageing was analyzed (including the non-aged sample as control) and three areas of 20 × 20 μm² per sample were recorded together with three areas of 5 × 5 μm². In order to estimate their surface roughness, the Root Mean Square roughness parameter (R_rms_) was calculated from 20 × 20 μm² topography images. The images were recorded in both height and phase mode. Visualization of the morphology was performed using the WSxM software [49]. Dimensions of protrusions and circular features were evaluated using the profile function of the VSxM software [49]. The estimation given was the mean value after evaluation of the sizes of each individual protrusions and circular feature on the different AFM images.

X-ray Photoelectron Spectroscopy analyzed the surface chemical composition. One sample per period of ageing was analyzed and three different spots were examined on each sample. These analyses were performed by using an X-ray Photoelectron Spectrometer (XPS-PHI 5600-ci Spectrometer-Physical Electronics, Eden Prairie, MN, USA). Charge compensation was not necessary. Survey and high resolution spectra were acquired using the Kα line of standard aluminium (Kα = 1486.6 eV) and magnesium (Kα = 1253.6 eV) X-ray sources respectively. Three different emission angles (θ) of 15 and 90° were applied, as measured from the surface plane, in order to obtain qualitative information about the variation of the chemical composition with depth. The curve fitting for the high resolution C (1s) peaks was determined by means of a least squares peak fitting procedure using Gaussian-Lorentzian functions (70–30% respectively) and a Shirley-type background. Five spectral components were used, representing the five major types of chemical environments of a carbon atom in a fluorocarbon structure: C-C/C-H (“neutral”or hydrocarbon), C-CF_x_, CF, CF and CF_3_. All components were assigned the same (adjustable) width (full width at half-maximum, FWHM). Their respective positions were not fixed. Assuming the binding energy (BE) of C-C/C-H to be 285.0 eV, the mean BEs obtained for the different C (1s) components were 286.0 eV (C-CF_x_), 288.8 eV (C-CF_x_), 291.4 eV (CF_2_) and 293.8 eV (CF_3_). These values, and the corresponding assignments, agreed well with data published in the literature [50]. However, as the film oxidized, these C (1s) signals would overlap with those resulting from the carbon-oxygen functionalities. In specific, the C (1s) signals of C-O, C=O, and O-C=O appear at binding energies of 286.5, 287.9, and 289.3 eV respectively, overlapping with the CCF_n_ and CFCF_n_ components. In a simplified protocol, the carbon-oxygen functionalities were not included in the C (1s) peak fitting since it is unlikely that a unique fit could be obtained with multiple C_x_F_y_O_z_ species [41].

Fourier Transform Infrared spectroscopy was performed with a Fourier Transform Infrared Spectrometer (Nicolet Magna 550, Thermo-Nicolet, Madison, WI, USA), equipped with a deuterated triglycine sulphate (DTGS) detector and a germanium coated potassium bromide beam splitter. The attenuated total reflection (ATR) mode was employed using split pea attachment (Harrick-Scientific Corp., Pleasantville, NY, USA) equipped with a silicon hemispherical internal reflection element. The depth of analysis was estimated to be 1 μm and 100 scans were routinely acquired at a spectral resolution of 4 cm^-1^. One sample per period of ageing was analyzed and three different spots were examined on each sample.

## 4. Conclusions

These experiments were carried out in the perspective to assess the potential for ageing into a pseudo-physiological medium to be considered as a first test to evaluate the stability of ultra-thin plasma polymerized coatings on stainless steel substrates. It was observed that the coating partially lost its hydrophobicity in the initial phase of the incubation process, which enabled buffer to penetrate throughout the film. A combination of XPS data at different probing depths and FT-IR results proved flat specimens to be covered with a metastable protective outermost layer, which initially inhibited degradation, but as a function of time started to dissolve into the ageing medium. Due to the plastic deformation procedure, the surface chemistry had undergone some compositional changes, which accelerated the ageing process of the films. In both cases degradation and oxidation of the ultra-thin fluorocarbon coating were observed. However, no metallic elements were detected by XPS, indicating that the coating protected and delayed the oxidation of the underlying stainless steel substrate.

Furthermore, the presence of nano-pinholes inducing water infiltration and therefore film degradation appeared to be a major problem. Further experiments in order to minimize the impact of these nano defects should be investigated. For example, a multi-layer formed, by modifying the plasma polymerization parameters during the deposition process, could break the connectivity of the inevitable defects and hence reduce their conductivity. The metastable oxidized layer evidenced by this study suggests that the formation of a controlled oxide layer on the top could also be a way to protect the film from degradation.
